# Tolerance to refractive error with a new extended depth of focus intraocular lens

**DOI:** 10.1038/s41433-024-03040-1

**Published:** 2024-04-05

**Authors:** Daniel A. Black, Chandra Bala, Aixa Alarcon, Srividhya Vilupuru

**Affiliations:** 1 Sunshine Eye Clinic, Birtinya, QLD Australia; 2 personalEYES, Paramatta, NSW Australia; 3 Johnson and Johnson MedTech, Groningen, The Netherlands; 4 Johnson and Johnson MedTech, Irvine, CA USA

**Keywords:** Medical research, Lens diseases

## Abstract

**Purpose:**

To evaluate the tolerance to refractive errors of a new purely refractive extended depth of focus (EDF) intraocular lens (IOL), TECNIS PureSee™ IOL, using preclinical and clinical metrics.

**Methods:**

Preclinical evaluation included computer simulations of visual acuity (sVA) and dysphotopsia profile of different IOL designs (refractive EDF, diffractive EDF, multifocal, standard, and enhanced monofocals) using an appropriate eye model with and without ±0.50 D defocus and/or +0.75 D of astigmatism. Patients bilaterally implanted with a refractive EDF (Model ZEN00V, TECNIS PureSee™ IOL) or an enhanced monofocal (Model ICB00, TECNIS Eyhance™ IOL) IOL from a prospective, randomized study were included. At the 6-month postoperative visit, uncorrected and corrected distance vision (UDVA and CDVA), visual symptoms, satisfaction and dependency on glasses were evaluated in a subgroup of patients with absolute residual refractive error of >0.25 D in one or both eyes.

**Results:**

In the presence of defocus and astigmatism, sVA was comparable for all except the multifocal IOL design. The refractive EDF was more tolerant to myopic outcomes and maintained a monofocal-like dysphotopsia profile with defocus. Binocular logMAR UDVA was −0.03 ± 0.08 for ZEN00V and −0.02 ± 0.11 for ICB00. 100% ZEN00V and 97% ICB00 patients did not need glasses and were satisfied with their distance vision. Monocular CDVA, contrast sensitivity and visual symptoms were also similar between both groups.

**Conclusions:**

The clinical outcomes of the refractive EDF IOL demonstrated high quality distance vision and dysphotopsia comparable to a monofocal IOL, even in the presence of refractive error, thus matching the design expectations of the EDF IOL.

## Introduction

Following cataract surgery, residual refractive errors can occur due to inaccuracies in IOL power calculations and/or biometric measurements [1, 2]. These postoperative refractive errors are common, may not be negligible in magnitude, and have been shown to be the most common cause of dissatisfaction following presbyopia-correcting IOL implantation [3]. In a study of 282,811 eyes, 73% of the eyes had a biometry prediction error within ±0.50 (dioptres) D, 93% were within ±1.0 D and the absolute mean prediction error in spherical equivalent was 0.42 D [4].

Multifocal IOLs separate light into different foci [5] and are much more sensitive to residual refractive errors compared to monofocal IOLs, which can impact patient satisfaction and quality of vision [3, 6, 7]. The reduction in quality of vision is associated with reduced contrast sensitivity and higher rates of halos and glare [2, 8, 9], which can be magnified in the presence of residual refractive error [3, 6, 7]. In the recent 2021 European Society of Cataract and Refractive Surgeons (ESCRS) clinical trends survey of 1570 physicians, the biggest concerns expressed against performing more presbyopia correcting IOL procedures, after cost to patient (58%), were night-time quality of vision (53%) and loss of contrast visual acuity (39%) [10]. In a retrospective chart review of 76 eyes of 49 patients following multifocal IOL implantation, de Vries et al. reported blurred vision (with or without photic phenomenon) in 72/76 eyes (94.7%) and photic phenomena (with or without blurred vision) in 29/76 eyes (38.2%). Both symptoms were present in 25/76 eyes (32.9%) [7].

Unlike multifocal IOLs, EDF IOL designs elongate the focal point to provide a continuous range of vision from distance to near [11, 12]. A large prospective post-market study in which patients were bilaterally implanted with the TECNIS^®^ Symfony diffractive EDF IOL (Johnson and Johnson Surgical Vision, Irvine, CA, USA) showed excellent distance vision with or without intended monovision correction, demonstrating the diffractive EDF IOLs ability to tolerate refractive error [13, 14]. IOL designs that provide more tolerance to refractive errors (TRE) can potentially benefit a large population of cataract patients. Practitioners often describe the TRE of IOLs following implantation as having a large ‘landing zone’ [15]; however, other factors such as distance image quality, may also be critical for visual performance.

The TECNIS^®^ PureSee^TM^ IOL (Johnson and Johnson Surgical Vision) has been developed to correct presbyopia utilizing a purely refractive EDF design. The IOL is designed to deliver a continuous range of vision, without compromising vision quality and contrast sensitivity while maintaining a dysphotopsia profile similar to that of a monofocal IOL, with increased TRE [16]. Currently, there is no standardized method established to evaluate and quantify TRE of IOLs. This paper will address both preclinical and clinical metrics related to demonstration of TRE in the TECNIS PureSee IOL. The preclinical metrics include computer simulations of uncorrected monocular distance visual acuity (sVA) and dysphotopsia in the presence of refractive errors for the TECNIS PureSee IOL as well as other IOL designs in the same TECNIS platform (Johnson and Johnson Surgical Vision). The clinical metrics included evaluation of uncorrected distance vision (UDVA), dependency on distance glasses, satisfaction with distance vision and reports of visual symptoms in a subgroup of subjects with residual ametropia following implantation with the TECNIS PureSee EDF IOL or the TECNIS^®^ Eyhance^TM^ enhanced monofocal IOL [17].

## Materials and methods

### Intraocular lenses

The test IOL in this study was the next generation refractive EDF TECNIS PureSee IOL (model ZEN00V, Johnson and Johnson Surgical Vision) and the control IOL was the enhanced monofocal TECNIS Eyhance IOL (model ICB00, Johnson and Johnson Surgical Vision). Details of the IOL design and surgical procedure have been published in a companion manuscript in this supplement [18].

### Preclinical methods

Computer simulations in a group of 46 physiological eye models with realistic corneas and higher-order aberrations [19, 20] were performed in white light with 3 mm pupils to calculate the computer sVA, using the radial averaged optical transfer function to account for rotational corneal asymmetries [21]. Simulations were performed with ±0.5 D of defocus and +0.75 D astigmatism correcting for the spherical equivalent. Additionally, computer simulations of a point light source in an average eye model [22] were performed in white light to illustrate the dysphotopsia profile in the presence of defocus. A large pupil of 5 mm was used to simulate low light conditions. The preclinical evaluation was performed for the model ZEN00V (test) and model ICB00 (control) IOLs, as well the standard monofocal TECNIS 1-piece (model ZCB00), the diffractive EDF TECNIS Symfony IOL (model ZXR00), and the TECNIS Multifocal IOL (model ZLB00).

### Clinical methods

#### Study design

A prospective, bilateral, randomized, subject and evaluator-masked comparative study was conducted in Australia and New Zealand (ClinicalTrials.gov; NCT04890249). Data collected from a total of six study sites were included. All patients provided written informed consent, and local independent human research ethics committee approval (Bellberry Limited, Human Research Ethics Committee and Health and Disability Ethics Committee) was obtained. The study was conducted in accordance with ISO 14155 Good Clinical Practice, the tenets of Declaration of Helsinki, and all other applicable laws and regulations of the countries in which the study was conducted. Subjects were bilaterally implanted with the TECNIS PureSee EDF IOL (test, model ZEN00V, *n* = 60) or the TECNIS Eyhance enhanced monofocal IOL (control, model ICB00, *n* = 57) and were followed for 6 months. Details of the study design, IOLs and surgical procedure have been published in a companion manuscript in this supplement [18]. To evaluate TRE, a subgroup analysis was conducted on patients in both the test and control groups that had absolute manifest refractive spherical equivalent (SEQ) greater than 0.25 D in one or both eyes at the 6-month postoperative visit.

Manifest refraction and visual acuity assessments were performed at 4 m, using the Early Treatment Diabetic Retinopathy Study (ETDRS) chart of the Clinical Trial Suite (CTS, M&S Technologies, Inc., Niles, IL USA) under photopic conditions (85–110 cd/m^2^). Monocular distance corrected contrast sensitivity was measured in first eyes at the 3-month visit under mesopic lighting conditions (3 cd/m^2^) both with and without glare. This was measured using the CTS system and sinewave grating charts encompassing frequencies of 1.5, 3, 6, and 12 cycles per degree (cpd) at 2.5 m; a refraction adjustment was used. Visual symptoms were evaluated using the validated Patient-Reported Visual Symptoms Questionnaire (PRVSQ) and satisfaction and spectacle use data were collected using the validated Patient Reported Spectacle Independence Questionnaire (PRSIQ).

#### Endpoints and assessments

In the subgroup of subjects, absolute SEQ, cylinder, binocular uncorrected distance visual acuity (UDVA), monocular best-corrected distance visual acuity (CDVA), monocular distance corrected mesopic contrast sensitivity with and without glare, frequency and bothersomeness of visual symptoms, the frequency of wear and need for glasses, and patient satisfaction with distance vision were assessed for the ZEN00V and ICB00 groups.

### Statistical analysis

To evaluate the tolerance to defocus, the percentage of model eyes that achieved 0.10 logMAR or better monocular uncorrected sVA was calculated in the presence of defocus and astigmatism. For the clinical data analysis, monocular refraction and visual acuity outcomes were reported by pooling the first and second eyes and monocular contrast sensitivity outcomes were reported for first implanted eyes only. Summary statistics included sample size (n), mean, and standard deviation (SD) for continuous variables. For categorical questionnaire data, the frequency and proportion were computed.

## Results

### Preclinical results

#### Simulated monocular uncorrected visual acuity

**Fig. 1:**
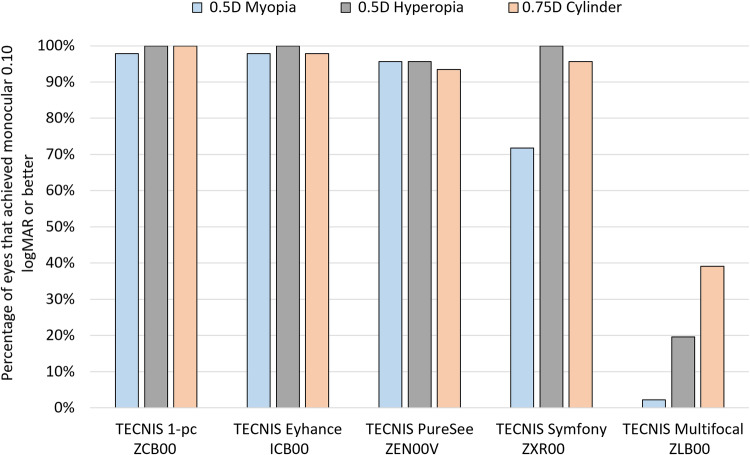
Percentage of eyes that achieved 0.10 logMAR or better monocular uncorrected simulated visual acuity (sVA). Results for test (ZEN00V) and control (ICB00) IOLs, and additional TECNIS platform IOL designs (ZCB00, ZXROO and ZLB00).

The percentage of eyes that achieved 0.10 logMAR or better monocular uncorrected sVA in the presence of defocus and astigmatism is presented in Fig. 1. These results show that the TECNIS PureSee IOL ZEN00V provides good distance visual acuity in >92% of eyes, at the level of the monofocal IOLs, TECNIS 1-piece and TECNIS Eyhance ICB00, and better than the TECNIS Multifocal IOL. Compared to the TECNIS Symfony, the refractive EDF provided the same high values for hyperopia and astigmatism but better results in the presence of myopia.

#### Dysphotopsia

**Fig. 2:**
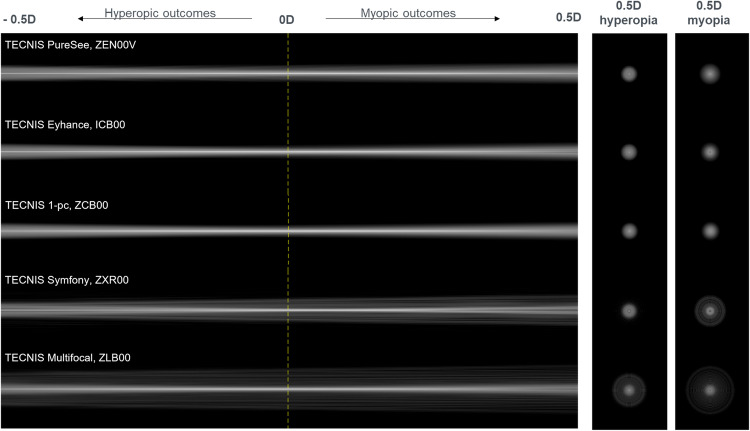
Dysphotopsia profile simulations from −0.5 D to +0.5 D of defocus simulated for a 5 mm pupil aperture with test (ZEN00V) and control (ICB00) IOLs, and additional TECNIS platform IOL designs (ZCB00, ZXROO and ZLB00). The vertical line illustrates the distance focus (0 D).

The effect of defocus on the dysphotopsia profile is illustrated in Fig. 2. These computer simulations of a point light source show that the ZEN00V IOL provides low levels of dysphotopsias comparable to the monofocal IOLs even in the presence of small amount of defocus (±0.50 D).

### Clinical results

The 6-month postoperative visit was completed by 60 ZEN00V and 57 ICB00 bilaterally implanted subjects with the study lenses. An analysis of the 6-month manifest refraction was conducted. Subjects that had absolute spherical equivalent (SEQ) greater than 0.25 D in one or both eyes were included in the subgroup evaluation. A total of 51.67% (31/60) bilateral subjects in the ZEN00V group and 50.88% (29/57) in the ICB00 group had an absolute SEQ > 0.25 D in one or both eyes at the 6-month visit.

#### Manifest spherical equivalent refraction and astigmatism

The mean absolute SEQ pooling the first and second eye data in the subgroup was 0.36 ± 0.19 D (*n* = 62 eyes) for the ZEN00V group and 0.46 ± 0.25 D (*n* = 58 eyes) for the ICB00 group. The mean absolute cylinder was 0.47 ± 0.30 D for the ZEN00V group and 0.47 ± 0.31 D for the ICB00 group. The mean absolute SEQ and cylinder were similar between the two groups.

#### Binocular uncorrected distance visual acuity (UDVA)

**Fig. 3:**
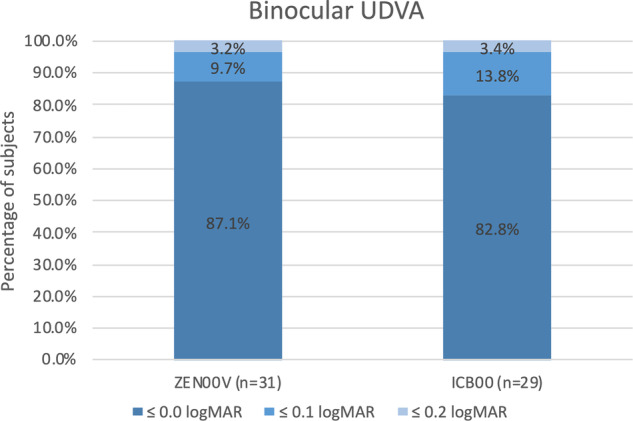
Percentage of patients with absolute SEQ of >0.25 D in one or both eyes achieving binocular uncorrected distance visual acuity (UDVA) of 0.0, 0.1 and 0.2 logMAR. Results for ZEN00V (test) and ICB00 (control) groups at 6 months.

Binocular UDVA was not significantly different between the groups at 6 months. The mean binocular UDVA was −0.03 ± 0.08 logMAR for the ZEN00V and −0.02 ± 0.11 logMAR for the ICB00 groups. As shown in Fig. 3, 87.1% (27/31) of ZEN00V and 82.8% (24/29) of ICB00 patients achieved 0.0 logMAR or better. 96.8% (30/31) of ZEN00V and 96.6% (28/29) of ICB00 patients achieved 0.1 logMAR or better. 100% of ZEN00V and ICB00 subjects achieved 0.2 logMAR or better at 6 months.

#### Monocular best-corrected distance visual acuity (CDVA)

Monocular CDVA was not significantly different between the groups. The mean monocular CDVA was −0.06 ± 0.07 logMAR for the ZEN00V and −0.06 ± 0.09 logMAR for ICB00 groups. Overall, 95.2% (59/62) of ZEN00V eyes and 86.2% (50/58) of ICB00 eyes achieved 0.0 logMAR or better. All (100%, 62/62) ZEN00V eyes and 98.3% (57/58) of ICB00 eyes achieved 0.1 logMAR or better. One hundred percent of ZEN00V and ICB00 eyes achieved 0.2 logMAR or better.

#### Monocular distance corrected contrast sensitivity

**Fig. 4:**
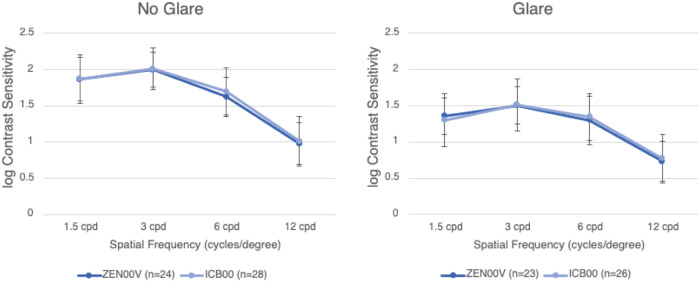
Mean monocular, distance corrected contrast sensitivity under mesopic lighting conditions without glare (left graph), and with glare (right graph) at the 3-month postoperative visit for ZEN00V and ICB00 first implanted eyes. Error bars represent ± standard deviation. Some data were not available from one site due to measurement error.

Figure 4 presents the monocular, distance corrected contrast sensitivity results at 3 months under mesopic conditions, with and without glare, in both ZEN00V and ICB00 groups. The mean values for contrast sensitivity were comparable between the ZEN00V and ICB00 IOL groups, with differences between the IOL groups falling within 0.07 log units under both conditions for all spatial frequencies.

#### Patient Reported Spectacle Independence Questionnaire (PRSIQ) for distance vision

None (0/31) of the patients in the ZEN00V group reported needing or wearing glasses for distance compared to 3.5% (1/29) in the ICB00 group. When asked to respond to how satisfied they were with their distance vision without glasses, the percentage of patients that reported being “completely satisfied” for distance vision was 93.5% (29/31) in the ZEN00V group compared to 82.8% (24/29) in the ICB00 group. The percentage of patients that reported being “mostly satisfied” for distance vision was 6.5% (2/31) for the ZEN00V and 17.2% (5/29) for the ICB00 groups. None of the patients reported being “moderately satisfied”, “a little satisfied” or “not at all satisfied” with their distance vision.

#### Visual symptoms

In the ZEN00V group, 87% (27/31), 100% (31/31), and 100% (31/31) of patients reported that they “never”, “rarely”, or “sometimes” experienced halos, starbursts and glare, respectively. In the ICB00 group, 93% (27/29), 93% (27/29), and 100% (29/29) of patients reported that they “never”, “rarely”, or “sometimes” experienced halos, starbursts and glare, respectively. Patients that reported experiencing halos, starburst or glare “often” or “always” were “not bothered” or “slightly bothered” in all cases except one ZEN00V patient, that reported “moderately bothered” for halos only.

## Discussion

Of the currently available presbyopia-correcting IOL technologies, surgeons report being most interested in integrating EDF IOLs into their practice [10]. However, concerns remain regarding vision quality, loss of contrast and the impact of not achieving emmetropia with presbyopia-correcting IOLs [6, 7, 10]. Modern EDF IOLs have been designed to address vision quality issues and improve the range of vision following surgery; however, current EDF IOLs can still lead to reduced contrast and dysphotopsias, which can worsen with residual refractive errors [2]. Since it is not possible to completely control all variables that lead to postoperative residual refractive errors following cataract surgery, an EDF IOL that provides an extended vision range with monofocal-like quality of vision and is more forgiving of residual refractive errors becomes critically important [5].

In this study, we evaluated the distance visual performance of ZEN00V, a new refractive EDF IOL designed to extend the depth focus and maintain monofocal-like visual quality, even in the presence of refractive errors. Simulated visual acuity with the ZEN00V IOL in the presence of ±0.50 D of defocus and +0.75 D of astigmatism demonstrated a high percentage of eyes achieving 20/25 or better (0.10 logMAR) uncorrected distance vision, which was similar to monofocal IOLs and significantly better than a multifocal IOL of the same platform.

The preclinical performance of the ZEN00V EDF IOL in the presence of defocus was supported by the visual performance of ametropic patients with the ZEN00V EDF IOL. Binocular UDVA in the ZEN00V group was similar to the ICB00 enhanced monofocal group (−0.03 logMAR vs. −0.04 logMAR, respectively). At 6 months, 87% of eyes achieved 0.0 logMAR binocular UDVA and 100% of patients were spectacle free for distance vision with the refractive ZEN00V EDF IOL. In comparison to another non-diffractive EDF IOL (AcrySof IQ Vivity, Alcon Inc, USA), the mean binocular UDVA was 0.07 ± 0.12 logMAR for AcrySof IQ Vivity compared to 0.03 ± 0.08 logMAR for AcrySof IQ monofocal IOL [23]. The mean spherical equivalent refraction was similar between the groups at −0.34 D for AcrySof IQ Vivity and −0.31 D for AcrySof monofocal IOL following surgery. Spectacle independence for distance vision was 80% with AcrySof IQ Vivity [23] compared to 100% with the ZEN00V EDF IOL in this study.

At 6 months, a reduction in mesopic contrast sensitivity, with and without glare, has been reported with the AcrySof IQ Vivity IOL at higher spatial frequencies compared to a monofocal IOL, which may explain the differences in performance when comparing the studies [24, 25]. In this study, mesopic contrast sensitivity with and without glare were comparable between the ZEN00V EDF and the ICB00 enhanced monofocal IOLs across the spatial frequency range. These results indicate that high quality distance vision contributes to increased TRE.

The simulated dysphotopsia profile data indicate that the new refractive EDF IOL provides a monofocal-like dysphotopsia profile in the presence of ±0.5 D of defocus. This result was confirmed by the clinical data which showed that patients implanted with the refractive EDF IOL with SEQ > 0.25 D had low incidence of halos, starburst, glare, similar to the enhanced monofocal IOL. The percentage of patients experiencing visual symptoms never, rarely, or sometimes in the SEQ > 0.25 D group was also comparable to that of the total cohort of patients, with 87% versus 88% for halos, 100% versus 97% for starbursts and 100% versus 100% for glare, respectively [18].

Patients are highly motivated to achieve spectacle independence following cataract surgery [11, 26, 27]. High levels of spectacle independence for distance vision were achieved in both groups. All patients with the ZEN00V refractive EDF IOL reported not needing glasses for distance vision, which compared favourably with another study of a diffractive EDF IOL, TECNIS Symfony IOL, which has shown high levels of tolerance to refractive errors, with 92.1% vs. 89.3% of patients that never/occasionally required spectacles for distance in a non-monovision and a monovision group with a target between 0.50 and 0.75 D respectively [13].

Although there was good alignment between the predicted performance of the new refractive EDF IOL in the presence of refractive errors from simulations and the clinical results, the clinical outcomes presented in this study are limited. During the clinical study, surgeons were requested to target emmetropia, thus the number of ametropic patients to include in the subgroup study were limited and had overall low levels of post-operative refractive errors. Future studies, in larger sample sizes or targeting monovision, could be conducted to further evaluate the effects of post-operative refractive errors and varying amounts of myopic offset on visual performance, dysphotopsias, and overall satisfaction.

In conclusion, this study has shown that the new refractive EDF test IOL provided high tolerance to small amounts of post-operative refractive errors, at the level of an enhanced aspheric monofocal IOL designed to slightly extend depth of focus. Excellent distance vision and contrast sensitivity, high patient satisfaction and a comparable dysphotopsia profile were demonstrated with the test IOL. The results of this study indicate that the tolerance to refractive errors of an intraocular lens could be driven by the combination of the extended depth of focus and high-quality distance vision.

## Summary

### What was known before

- Multifocal IOLs are much more sensitive to residual refractive errors than monofocal IOLs, which can impact patient satisfaction and quality of vision.
- Extended depth of focus (EDF) IOL designs provide a continuous range of vision from distance to near, but diffractive EDF designs can still result in reduced contrast sensitivity and higher rates of halos and glare.
- EDF IOLs are more tolerant to residual errors compared to multifocal IOLs.

### What this study adds

- The novel purely refractive EDF IOL provided high quality distance vision and a dysphotopsia profile comparable to a monofocal IOL, even in the presence of residual refractive error.
- Presbyopia-correcting IOL designs that provide greater tolerance to refractive errors can potentially benefit cataract patients and alleviate surgeons’ concerns regarding quality of vision and loss of contrast visual acuity with refractive misses.

## Data Availability

The authors do not intend to share individual deidentified participant data. A summarized report with endpoints data tables based on statistical plan and analysis may be requested directly from the corresponding author for consideration. Access to anonymized data may be granted following review. Content with granted access will be available through email or other appropriate formats and for 3 months, upon review and consideration.
