# Correction: Tolerance to refractive error with a new extended depth of focus intraocular lens

**DOI:** 10.1038/s41433-024-03422-5

**Published:** 2024-11-05

**Authors:** Daniel A. Black, Chandra Bala, Aixa Alarcon, Srividhya Vilupuru

**Affiliations:** 1 Sunshine Eye Clinic, Birtinya, QLD Australia; 2 personalEYES, Paramatta, NSW Australia; 3 Johnson and Johnson MedTech, Groningen, The Netherlands; 4 Johnson and Johnson MedTech, Irvine, CA USA

**Keywords:** Medical research, Lens diseases

Correction to: *Eye* 10.1038/s41433-024-03040-1, published online 05 April 2024

The ‘PURPOSE’ section of the abstract was corrected from “To evaluate the tolerance to refractive errors of a new purely refractive extended depth of focus (EDF) intraocular lens (IOL) using preclinical and clinical metrics” to “To evaluate the tolerance to refractive errors of a new purely refractive extended depth of focus (EDF) intraocular lens (IOL), TECNIS PureSee™ IOL, using preclinical and clinical metrics.” Additionally, in the ‘METHODS’ section of the abstract, the second sentence was corrected from “Patients bilaterally implanted with a refractive EDF (Model ZEN00V) or an enhanced monofocal (Model ICB00) IOL from a prospective, randomized study were included” to “Patients bilaterally implanted with a refractive EDF (Model ZEN00V, TECNIS PureSee™ IOL) or an enhanced monofocal (Model ICB00, TECNIS Eyhance™ IOL) IOL from a prospective, randomized study were included.” The original article has been corrected.

